# Use of Electronic Messaging and the News Media to Increase Case Finding During a *Cyclospora* Outbreak — Iowa, July 2013

**Published:** 2013-08-02

**Authors:** Nicholas Kalas, Patricia Quinlisk

**Affiliations:** Iowa Dept of Public Health

On Friday, June 28, 2013, the Iowa Department of Public Health (IDPH) routinely reported two cases of cyclosporiasis in its weekly electronic newsletter, the *EPI Update.* The newsletter’s primary audience consists of Iowa’s public health officials and health-care providers, but readers also include members of the news media.

By Wednesday, July 3, an additional four cases had been reported to IDPH, indicating that an outbreak could be occurring (before 2013, only 10 cases had been reported in Iowa). In response, IDPH released a special *EPI Update Alert* and a Health Alert Network alert to all hospitals, emergency departments, infection preventionists, public health agencies, and other health-care providers in Iowa. Both electronic alerts included information on symptoms of cyclosporiasis, and diagnosis and treatment guidelines. By July 4, when a CDC Epidemic Information Exchange alert was issued, most major media outlets in Iowa had reported on the outbreak. An e-mail press release with updated information was issued on July 8 to nearly 400 members of the news media, and the first round of 14 messages was sent to 5,282 Twitter followers. By July 9, daily updates, including case counts, were being requested by the media and posted on the IDPH website. Over the next several weeks, as health-care providers and the public became aware of the outbreak, many Iowans were tested and given diagnoses of cyclosporiasis.

*Cyclospora*, a coccidian parasite, can cause prolonged and relapsing watery diarrhea, which, if untreated, can last several weeks to months (1). This long duration of diarrhea is unusual among foodborne pathogens and allows time for patients to seek medical attention and have their illness diagnosed (1,3). Testing for *Cyclospora* is not routinely done in most U.S. laboratories, even when stool specimens are tested for parasites; health-care providers must specifically request testing for *Cyclospora* when indicated. The preferred treatment for cyclosporiasis is trimethoprim-sulfamethoxazole; to date, no reliably effective alternative treatments have been identified (1,3). *Cyclospora* is not spread from person to person because excreted oocysts require several days to weeks outside the host to become infectious. Previous outbreaks in the United States have been associated with fruits (i.e., raspberries [2]) and raw vegetables (i.e., mesclun lettuce, basil, and snow peas [3]).

As of July 26, nearly all of Iowa’s 135 reported cases of cyclosporiasis had been diagnosed by testing at the state’s public health laboratory, the State Hygienic Laboratory (SHL) (Figure). The two initial cases, reported in the June 28 issue of *EPI Update*, were diagnosed by a laboratorian at SHL who identified possible *Cyclospora* oocysts on microscopic examination of fresh stool and confirmed this diagnosis using a modified acid-fast stain technique. In June at SHL, before electronic messaging was used and media attention was attracted, 271 stool tests for ova and parasites were requested but none specifically for *Cyclospora*. In contrast, during the first 23 days of July, requests for general ova and parasite stool tests had increased to 762, and specific *Cyclospora* testing was requested on 1,460 specimens.

In addition to case ascertainment, increased attention by the news media and health department notifications led to improved diagnosis and treatment. For example, one patient with severe vomiting and diarrhea was discharged without a diagnosis after a 5-day hospital stay and extensive laboratory testing, only to relapse days later. After reading the *EPI Update Alert,* the patient’s health-care provider ordered *Cyclospora* testing on the patient, and the result was positive. The patient was treated with trimethoprim-sulfamethoxazole, and the symptoms resolved.

The use of electronic messaging and media attention in the early stages of this outbreak investigation provided public health agencies a unique opportunity to increase testing for this uncommon disease, which might not otherwise have been considered by health-care providers or their patients. After diagnosis and case reporting, patients provided IDPH with valuable information on their potential exposures to *Cyclospora*, increasing the power of statistical analyses and the likelihood of finding the source of the infection. The *Cyclospora* outbreak investigation is ongoing.

## Figures and Tables

**FIGURE:**
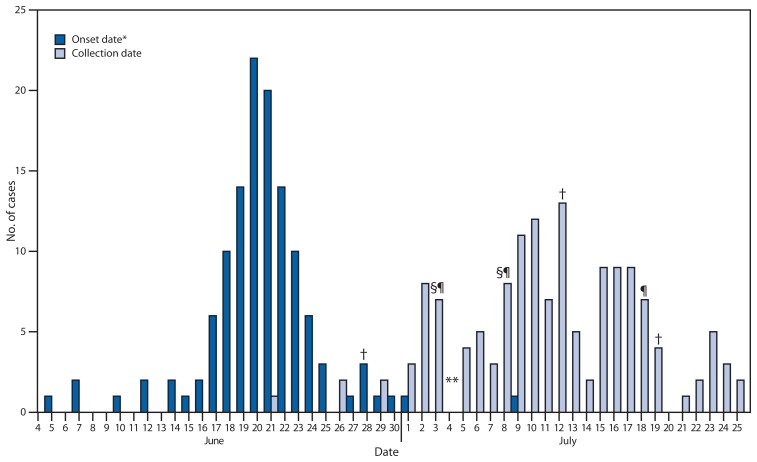
Number of confirmed cases of cyclosporiasis (N = 135), by dates of symptom onset and collection of laboratory specimen, and dates of electronic health alerts — Iowa, June 4–July 25, 2013 * Illness onset date was not available for 11 confirmed cases. ^†^ Iowa *EPI Update*. ^§^ Iowa EPI Update Alert. ^¶^ Iowa Health Alert Network alert. ** CDC Epidemic Information Exchange alert.
